# 

**Published:** 1891-10-10

**Authors:** 


PRICE ONE PENNY.

No. 263, Vol. XI.—October 10th, 1891.

[Registered at the General Post Office as a Newspaper for transmission in the United Kingdom and Abroad.]

WITH EXTRA NURSING SUPPLEMENT.

Per Annum, 6s. 6d.; post free.

Monthly Parts, 6d.; post free 8d.

Ditto per Annum, 8s., post free.

### THE HOSPITAL

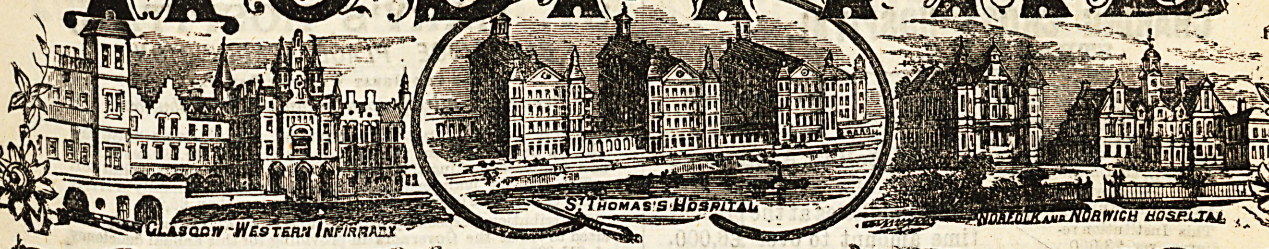

A WEEKLY JOURNAL OF

### Science, Medicine, Nursing and Philanthropy.

### SATURDAY, OCTOBER 10th, 1891.

**Confusion Worse Confounded** . . . 13

**Passing Topics.**—Shall Nurses Cease to be Laywomen?—V. . . . 14

**The Doctor's Armchair.**—Scientific Scoundrelism . . . 15

**Liverpool Royal Infirmary.** By Our Special Commissioner 16

**Editor's Letter-Box.**—Antiseptic Treatment of Scarlet Fever 16

**Talks About Food.** I.—The Relation of Food to Labour . 17

**Notes and News** . . . 18

**General Charities** . . . 19

**Scraps and Gleanings** . . . 19

**The Need for Female Factory Inspectors** . . . 24

**Annotations.**—Hospital Religion—Our Unseen Foes—Folklore in Congress . . . 20

**The Practitioner's Mirror and Retrospect**—

MEDICINE.—New Methods of Treatment in Phthisis. II.—The Gibbes-Shurly Method . . . 21

SURGERY.—On Some Affections of the Thyroid . . . 22

PRACTITIONERS' BOOKSHELF.—The Intubation of the Larynx—On Stertor, Apoplexy, and the Management of the Apoplectic State . . . 23

**The Student of Medicine.**—Appointments—Vacancies—Scholarships and Prizes.

### EXTRA SUPPLEMENT—THE NURSING MIRROR.

**En Passant.**—Short Items—The Hamilton Association—Six Months' Certificates—Lincoln Nurses—Mr. James Payne on Nurses . . . vii

**On Private Nursing** . . . viii

**A Bed for a Sick Nurse** . . . viii

**The Princess of Wales and the Nurses** . . . ix

**Where to Go—Love Divine** . . . ix

**For Reading to the Sick**—God's Promise Point d Out . . . ix

**On Things in General, and Massage in Particular** . x

**Everybody's Opinion.**—Objectionable Advertisements—Nurses as Patients, x.; An Appeal for Dolls—Monthly Nurses—Six Months' Certificates . . . xi

**Examination Questions** . . . xi

**Notes and Queries** . . . xi

**Off Duty.**—Kitty's Find—Home Again . . . xii

**Amusements and Relaxation** . . . xii

